# Supplementary material for: A Global Screen for Assembly State Changes of the Mitotic Proteome by SEC-SWATH-MS
Source: Cell Syst. 2020 Feb 26;10(2):133–155.e6. doi: 10.1016/j.cels.2020.01.001 (PMC7042714; doi:10.1016/j.cels.2020.01.001)

Q7Z3K3 | POGZ_HUMAN | POGZ KIAA0461 SUHW5 ZNF280E ZNF635 Nbla00003
Monomer MW [kDa]: 155.344 Monomer expected elution fraction: 36

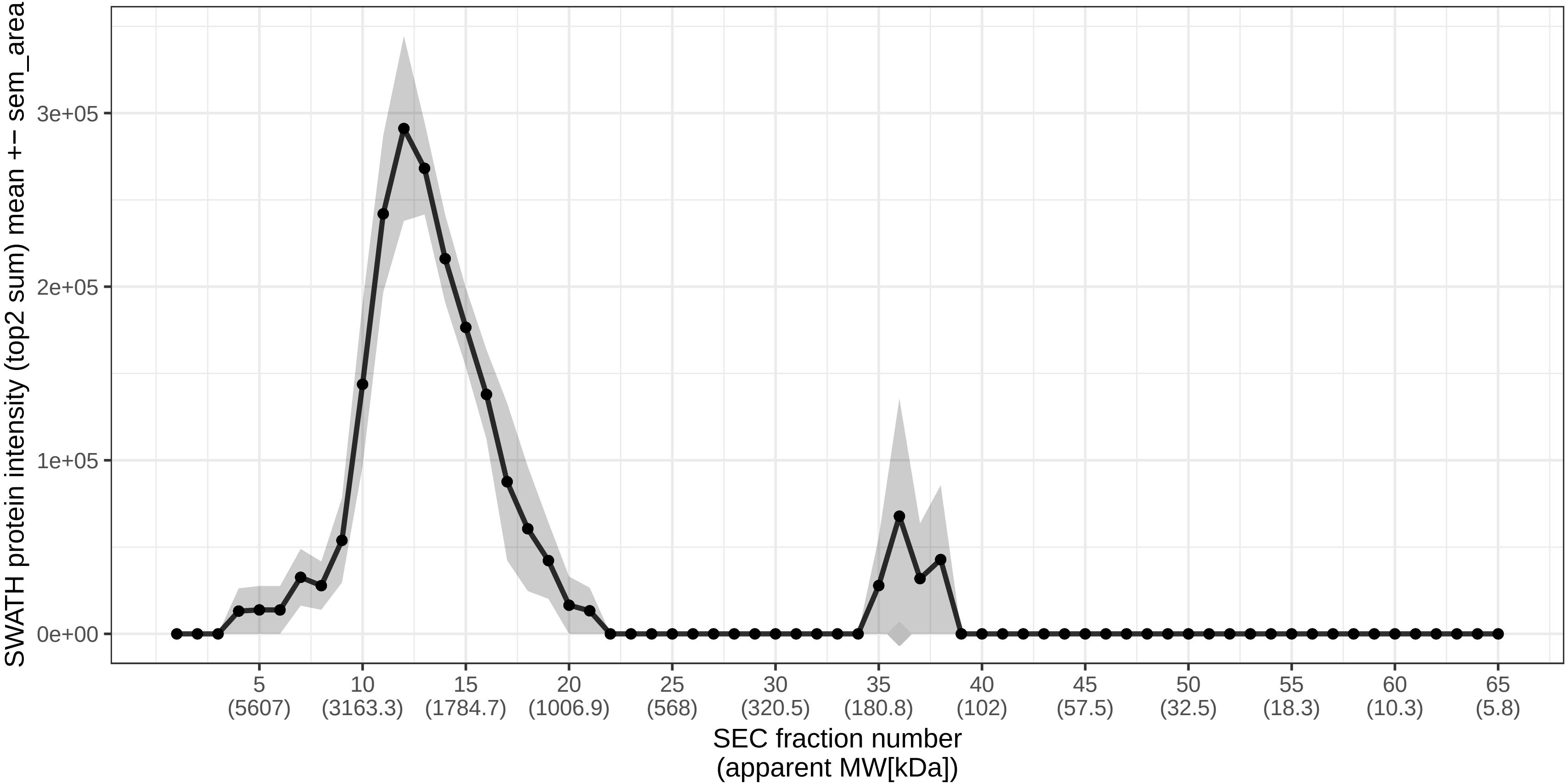

Supplement: Data S1. SEC-SWATH-MS Protein Chromatograms, Related to Figure 1 [file mmc6.zip › SECchrom_Q7Z3K3_POGZ_HUMAN_POGZ_KIAA0461_SUHW5_ZNF280E_ZNF6.pdf]
